# Hyperhidrosis quality of life index (HidroQoL): further validation by applying classical test theory and item response theory using data from a phase III clinical trial

**DOI:** 10.1186/s41687-023-00596-6

**Published:** 2023-06-06

**Authors:** Theresa Donhauser, Christian Apfelbacher, Gesina Kann, Clarissa Masur, Paul Kamudoni, Sam Salek, Christoph Abels, Michaela Gabes

**Affiliations:** 1 Institute of Social Medicine and Health Systems Research, Otto-von-Guericke University Magdeburg, Leipziger Str. 44, 39120 Magdeburg, Germany; 2 University of Regensburg, Regensburg, Germany; 3 Dr. August Wolff GmbH & Co. KG Arzneimittel, Bielefeld, Germany; 4 Darmstadt, Germany; 5 School of Life and Medical Sciences, University of Hertfordshire, Hertfordshire, UK; 6 Institute of Medicines Development, Cardiff, UK

**Keywords:** Hyperhidrosis, Hyperhidrosis quality of life index, Quality of life, HidroQoL, Patient-reported outcome measure, Structural validity

## Abstract

**Background:**

The Hyperhidrosis Quality of Life Index (HidroQoL ©) is a well-developed and validated patient-reported outcome measure assessing the quality-of-life impacts in hyperhidrosis with 18 items. Our aim was to extend the already existing validity evidence for the HidroQoL, especially in relation to structural validity. Especially Rasch analysis has not been applied to the final 18-item HidroQoL before.

**Methods:**

Data from a phase III clinical trial were used. Confirmatory factor analysis was conducted to confirm the two a priori HidroQoL scales within classical test theory. Furthermore, the assumptions of the Rasch model (model fit, monotonicity, unidimensionality, local independence) and Differential Item Functioning (DIF) were assessed using item response theory.

**Results:**

The sample included 529 patients with severe primary axillary hyperhidrosis. The two-factor structure could be confirmed by the confirmatory factor analysis (SRMR = 0.058). The item characteristic curves showed mainly optimally functioning response categories, indicating monotonicity. The overall fit to the Rasch model was adequate and unidimensionality for the HidroQoL overall scale could be confirmed, since the first factor had an eigenvalue of 2.244 and accounted for 18.7%. Local independence was below assumed thresholds (residual correlations ≤ 0.26). DIF analysis, controlling for age or gender, was critical for four and three items, respectively. However, this DIF could be explained.

**Conclusion:**

Using classical test theory and item response theory/Rasch analyses, this study provided further evidence for the structural validity of the HidroQoL. This study confirmed several specific (measurement) properties of the HidroQoL questionnaire in patients with physician-confirmed severe primary axillary hyperhidrosis: the HidroQoL is a unidimensional scale allowing the summation of scores to generate a single score, and simultaneously it has a dual structure, also allowing the calculation of separate domain scores for daily activities and psychosocial impacts. With this study, we provided new evidence of the structural validity of the HidroQoL in the context of a clinical trial.

*Trial registration* The study was registered (ClinicalTrials.gov identifier: NCT03658616, 05 September 2018, https://clinicaltrials.gov/ct2/show/NCT03658616?term=NCT03658616&draw=2&rank=1).

## Background

Hyperhidrosis (HH) is a clinical condition causing excessive sweating that exceeds the physiological needs of the person concerned [1]. This condition can either be classified as primary HH due to an overactivity of the sympathetic nerves or as secondary if the excessive sweating results from a medical condition or the consumption of medications [2]. In the US, approximately 2.8% of the population are affected by this condition, half of which suffer from axillary hyperhidrosis. Furthermore, more than 10% of four million individuals affected by axillary HH rated their disease as intolerable and stated that it interferes with their day to day activities [3]. HH can range from dampness of parts of the body to severe dripping and therefore, this condition possibly has a substantial impact on the patient’s life [2] and can be detrimental to the patients’ social, psychological, professional, and physical well-being [4].

These individual impacts can be captured using Patient-Reported Outcome Measures (PROMs) which are self-completed questionnaires capturing the individual perspective of the patients themselves rather than their physicians. As there are many PROMs regarding hyperhidrosis, Gabes et al. [5] conducted a systematic review of the quality of existing PROMs. As a result, three PROMs were rated as category A meaning that these questionnaires have sufficient measurement properties and that they can be recommended for future use. These three PROMs were the Hyperhidrosis Questionnaire (HQ) [6], the Sweating Cognitions Inventory (SCI) [7] and the Hyperhidrosis Quality of Life Index (HidroQoL) [8, 9]. Of these three PROMS, the HidroQoL proved to be the most convincing in the systematic review, as it had a higher level of evidence for content validity (moderate) and internal consistency (high) than the HQ and SCI. Its strong measurement properties were also supported in terms of structural validity, reliability, construct validity, and responsiveness, all of which received sufficient ratings and high-quality evidence and were based on larger study populations. In this study, we focused on the HidroQoL and aimed to evaluate its psychometric properties (especially the structural validity) in patients with primary axillary HH, thereby extending existing validity evidence [9, 10]. Modern test theory, especially Rasch analysis, has not been performed on the final 18-item HidroQoL before.

## Patients and methods

In a phase III (a/b) clinical trial, patients with primary axillary HH were asked to complete the HidroQoL at several timepoints (baseline, after 4, 8, 12, 28, 52, and 72 weeks). This clinical trial investigated the effects of a topical cream containing 1% glycopyrronium bromide for which safety and efficiency was reported recently [11]. Ethical approval was obtained by the corresponding ethics committees of the different countries and the study was registered (ClinicalTrials.gov identifier: NCT03658616). It was a multi-national (UK, Sweden, Denmark, Germany, Poland and Hungary), multi-center (*n* = 37) trial [11]. The study was sponsored by Dr. August Wolff GmbH & Co. KG Arzneimittel.

Data from this clinical trial (phase III a) have already been used for previous validation analyses [10]. In the validation analyses of this manuscript, the baseline (pooled) data of the phase III b clinical trial was used for the assessment of structural validity since high sample sizes are required when performing Rasch analysis. The manuscript was prepared in accordance with the COnsensus-based Standards for the selection of health Measurement INstruments (COSMIN) Reporting guideline for studies on measurement properties of PROMs (Appendix 1) [12].

### The HidroQoL

The HidroQoL was developed in 2014 with qualitative patient and expert input. An 18-item questionnaire with three response options resulted. Following qualitative development, the initial validation was based on two observational studies. Overall, the instrument showed very good measurement properties supporting its use in clinical practice in order to assess the impact of HH on Quality of Life (QoL) [8–10].

**Fig. 1:**
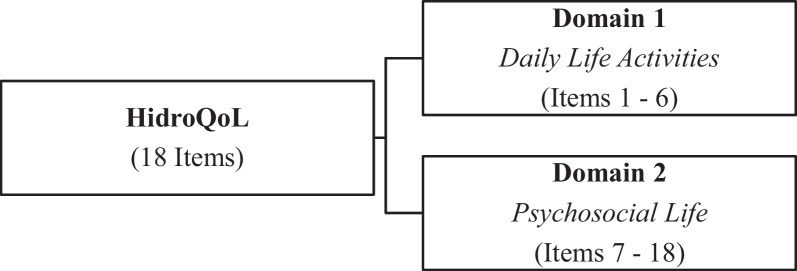
Main domains of the Hyperhidrosis Quality of Life Index (HidroQoL)

For this reason, the HidroQoL is a well-developed and validated PROM, which measures the QoL impacts in HH. With two main domains and 18 items in total, the questionnaire is short enough to exclude irrelevant topics but still is able to comprehensively assess the impact that HH has. The first domain with six items evaluates the impact of the condition on daily life activities (such as hobbies). The second domain captures the psychosocial life of the affected individuals (such as personal relationships) (see Fig. 1). The participants can choose between three response options (0: No, not at all; 1: A little; 2: Very much). The items are considering the past seven days. The total score ranges from 0 to 36. Before the clinical trial started, different language versions of the HidroQoL have been linguistically validated including forward–backward-translations and cognitive debriefing.

### Data analysis

Psychometric analyses based on classical test theory (CTT) and item response theory (IRT) analyses including Rasch analysis were performed to evaluate the structural validity and other psychometric properties of the HidroQoL. All statistical analyses were performed using IBM SPSS Statistics 25, MPlus 8.4 software (*Muthen* & *Muthen*, *Los Angeles, CA*), SAS 9.4 and Winsteps.

### Distribution of responses

In order to evaluate whether data is missing at random, the pattern of missing data was assessed. Furthermore, we investigated floor and ceiling effects.

### Using CTT: confirmatory factor analysis

Confirmatory factor analysis (CFA, using maximum likelihood as an estimator) was performed to verify the two a priori scales of the HidroQoL. According to the COSMIN initiative, the structural validity of an instrument is rated as sufficient if one of the following requirements is met: either the Comparative Fit Index (CFI) or Tucker–Lewis Index (TLI) is > 0.95 or the Root Mean Square Error of Approximation (RMSEA) is < 0.06 or the Standardized Root Mean Square Residual (SRMR) is < 0.08 [13].

### Using IRT: analysis of the response categories and performing Rasch analysis

We also performed Rasch analysis in order to confirm unidimensionality, and to determine the model fit, the monotonicity, the local independence and the Differential Item Functioning (DIF). In general, a minimum of ten observations per category per item is recommended in order to reach a stable estimation of category thresholds [14]. It is necessary to include a large and heterogeneous sample of patients reflecting varying levels of disease severity (based on their Hyperhidrosis Disease Severity Scale (HDSS)-Score). If this requirement is fulfilled, it is ensured that the respondents reflect the entire continuum of the construct (from the highest possible QoL impairment to the minimum possible impairment). Therefore, it was aimed to recruit a sample of at least 243 participants in order to achieve precision even in heavily skewed data [14, 15].

According to the COSMIN criteria for a sufficient structural validity rating the following requirements must be fulfilled: an adequate model fit, no violation of monotonicity, unidimensionality and local independence [13].

### Model fit

Model fit was assessed for the entire scale, the individual items, and the persons. An overall model fit is reflected by a mean fit residual value of 0 and a standard deviation (SD) of 1–1.5 [15, 16]. For the individual item and person level, infit and outfit mean squares were analyzed. These should be ≥ 0.5 to avoid overfit (redundancy) and ≤ 1.5 to avoid underfit (too much measurement error) [13]. To check the adequacy in spread of the items along the breadth of the latent variable the item-person map was visually examined. Ideally, there should be no large gaps between items [17] and the mean location of persons should be close to 0 to match the item mean location centered at 0 logits [18]. Furthermore, the Person Separation Index (PSI) was calculated to assess the ability of the instrument to differentiate persons according to disease severity. Here, a PSI of 0.8 reflects capability to reliably distinguish patients into at least two groups of severity [17, 19].

### Monotonicity

We created item characteristics curves (ICCs) in order to assess the functioning of the response options. Here, the category thresholds should monotonically increase with the category and each response category should have a distinct peak on the graph [14, 15].

### Unidimensionality

Unidimensionality was assessed conducting a principal component analysis (PCA) on the residuals of the Rasch model regression. For unidimensionality, the first factor must not account for more than 30% of the variance in the data and must have an eigenvalue of 3 or less [14]. Furthermore, unidimensionality refers to a factor analysis per subscale. Therefore, the CFA was carried out for the HidroQoL as a single scale. Unidimensionality is not violated if the CFI or TLI is > 0.95 or the RMSEA is < 0.06 or the SRMR is > 0.08 according to the COSMIN criteria [13].

### Local independence

For testing the local independence, the correlation matrix of the item residuals was examined. A violation of this assumption is reflected by residual correlations exceeding 0.2–0.3 [15, 20].

### Differential item functioning (DIF)

DIF was assessed for the key demographic factors gender and age using a two-way ANOVA test. DIF by country was not assessed given the huge difference in sample sizes (e.g. Germany: *n* = 156 vs. United Kingdom: *n* = 10). Invariance testing on small sample sizes was considered as problematic. For a significant DIF on an item the probability must be ≤ 0.05 and the difference in the item difficulty must exceed 0.43 logits. Based on these thresholds, we used the following categorization of DIF sizes (Table 1) [21].

**Table 1:** Categorization of the DIF sizes

DIF category	Category explanation	DIF contrast
C	Moderate to large	|DIF| ≥ 0.64 logits
B	Slight to moderate	|DIF| ≥ 0.43 to < 0.64 logits
A	Negligible	|DIF| < 0.43 logits

*DIF* Differential item functioning

## Results

The sample consisted of *n* = 529 participants with severe primary axillary hyperhidrosis, represented by a HDSS score of 3 or 4. Of these, 283 of the patients were female (53.5%) and 246 were male (46.5%). The mean age of the study participants was 35.61 years (SD = 11.68), with a median of 33 years. The age range was from 18 to 65 years.

### Distribution of responses

Only item 18 had a single missing entry at baseline. The test for normal distribution over the subjects' sum scores was significant, indicating a left-skewed distribution with some ceiling effects. These effects can be explained as a result of the homogeneous study population, which included only patients with severe hyperhidrosis. The percentage of participants selecting the highest response option (very much) across the items ranged from 16.8 to 88.7%.

### Using CTT: confirmatory factor analysis

Confirmatory factor analysis confirmed the *a-priori* assumed two-factor structure of the HidroQoL. With a value of 0.058, SRMR fulfilled the COSMIN criteria and thus supported sufficient structural validity. Other key values are listed in Table 2.

**Table 2:** Goodness-of-fit indices obtained by the confirmatory factor analyses at baseline (*n* = 528)

Goodness-of-fit index		Two-factor structure	COSMIN criteria
CFI	Comparative fit index	0.857	≥ 0.95
TLI	Tucker–Lewis index	0.837	≥ 0.95
RMSEA	Root mean square error of approximation	0.086 (90% CI 0.080–0.093)	< 0.06
SRMR	Standardized root mean square residual	0.058	< 0.08

*CI* Confidence interval, *COSMIN* COnsensus-based Standards for the selection of health Measurement INstruments

### Using IRT: analysis of the response categories and performing Rasch analysis

#### Model fit

**Fig. 2:**
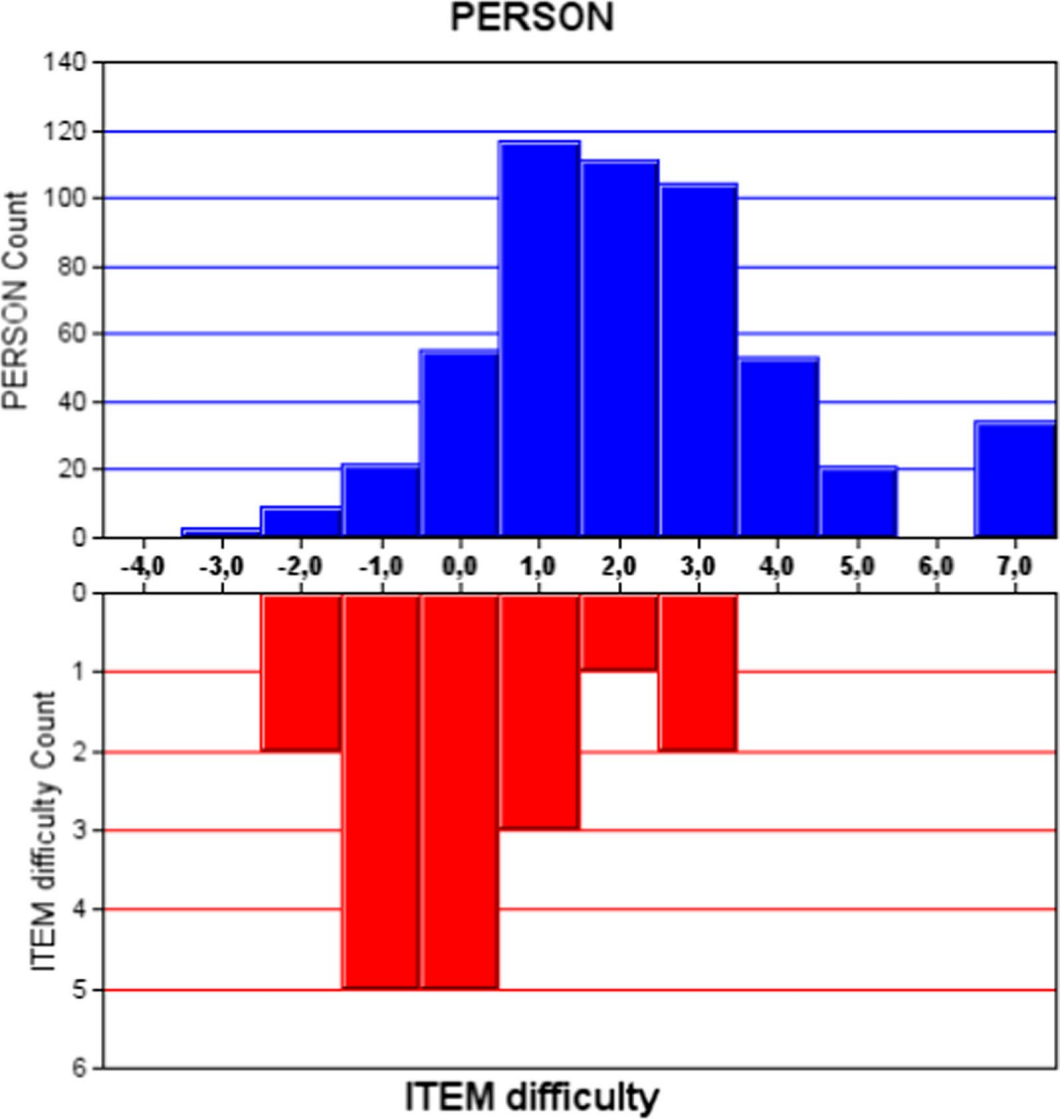
Person-item map

Overall model fit was adequate with a mean fit residual of 0 (SD = 1.37). The mean squares of infit and outfit presented in Table 3 were above 0.5 (infit) and below 1.5 (outfit) for all items, indicating that the items are not redundant with each other. The correlations and expected correlations were close to each other. Thus, an adequate model fit for the HidroQoL overall scale was given. Visual examination using the person-item map (Fig. 2) revealed an adequate spread of item difficulty centered around zero on the scale. The plot of the person measures, however, reflected the left-skewed distribution of the data. Person measures and item difficulty were thus slightly shifted against each other along the scale, which implies that persons with higher severity of hyperhidrosis in our sample might not be well differentiated by the questionnaire.

**Table 3:** Infit and outfit mean square for the 18 items of the HidroQoL (Baseline)

Items	Infit MNSQ	Outfit MNSQ	Items	Infit MNSQ	Outfit MNSQ
Item 1	0.95	1.11	Item 10	0.99	0.95
Item 2	0.96	0.92	Item 11	1.03	1.05
Item 3	0.88	0.89	Item 12	0.98	1.02
Item 4	1.09	0.95	Item 13	0.94	0.75
Item 5	0.93	1.08	Item 14	1.33	1.20
Item 6	0.85	0.84	Item 15	0.80	0.79
Item 7	1.13	1.20	Item 16	1.15	1.13
Item 8	1.09	0.93	Item 17	0.85	0.90
Item 9	1.17	1.13	Item 18	0.95	1.01

*HidroQoL* Hyperhidrosis quality of life index, *MNSQ* Mean squares

#### Monotonicity

**Fig. 3:**
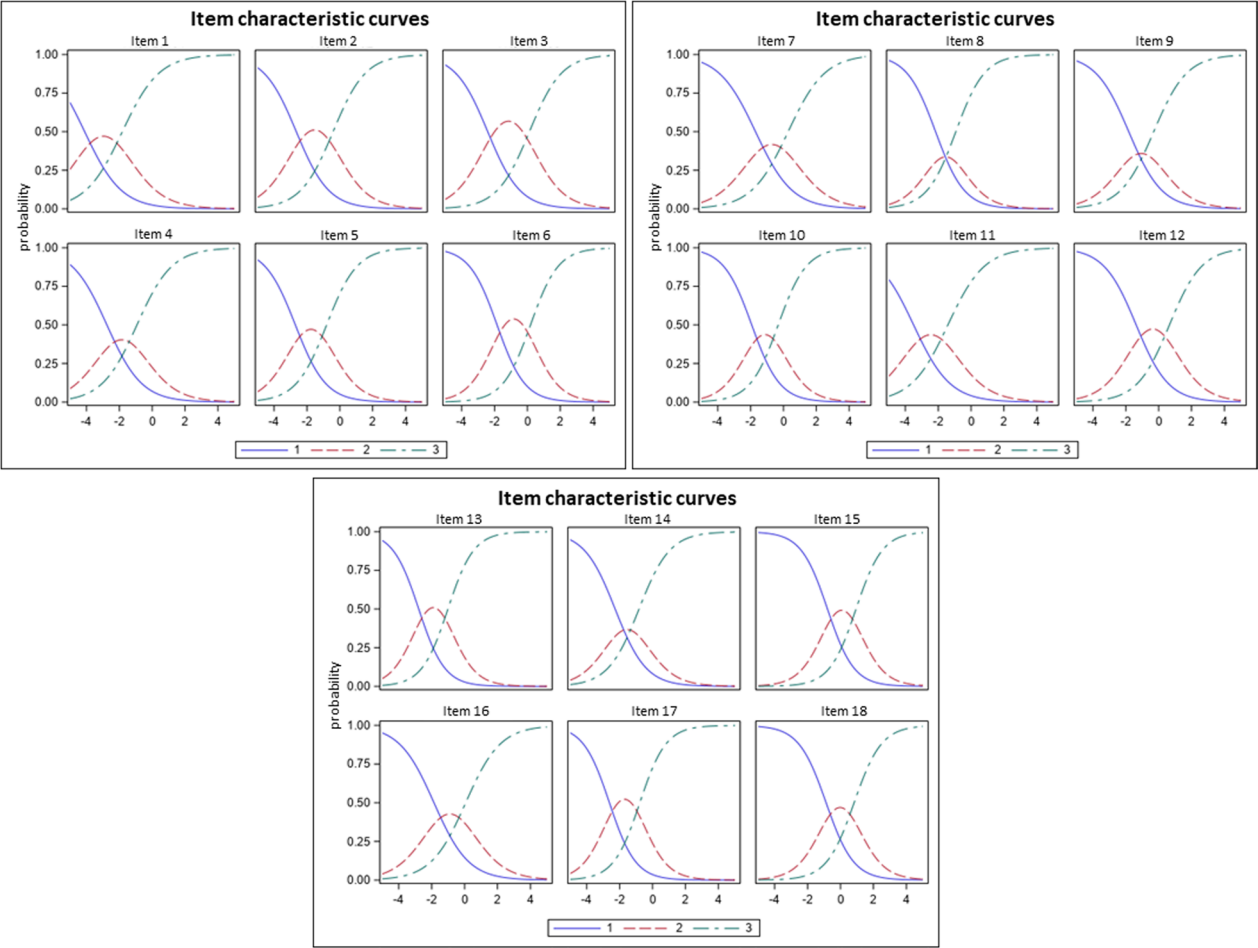
Item characteristic curves for items 1–18

All items of the HidroQoL showed adequate looking graphs, indicating optimally functioning response categories (Fig. 3). However, it should be noted that for both item 1 (“My choice of clothing is affected”) and item 10 (“I feel uncomfortable physically expressing affection (e.g. hugging)”), the recommended minimum number of ten observations per response category was not reached for the lowest category in either case. Item 8 (“I feel embarrassed”) is slightly ambiguous, as the middle response category could not be assigned a distinct range along the scale. Overall, the criterion of monotonicity can be assumed.

#### Unidimensionality

The criterion of unidimensionality was fulfilled, as demonstrated by the PCA of the residuals. The first component of the PCA had an eigenvalue of 2.244 and accounted for 18.7% of the variance. In addition, structural validity was demonstrated by confirmatory factor analysis for the HidroQoL overall scale. With a CFI of 0.811, a TLI of 0.786, an RMSEA of 0.099, and an SRMR of 0.063, the scale met at least one COSMIN criterion for sufficient structural validity.

#### Local independence

Regarding local independence, residual correlations were above 0.2 in five cases, however, above 0.3 in no case. Correlations and corresponding items are shown in Table 4.

**Table 4:** Largest standardized residual correlations

Residual correlation	Items
0.26	Item 2 & Item 3
0.24	Item 15 & Item 16
0.23	Item 3 & Item 6
0.23	Item 2 & Item 6
0.23	Item 8 & Item 9

In terms of reliability, the HidroQoL achieved a PSI of 0.85 and showed a person separation of 2.36, meaning that the PROM was able to differentiate between at least two statistically significantly different severity groups. Item separation had a value of 12.99 with a high item reliability of 0.99, reflecting almost 13 levels of item difficulty in the data. Thus, the difficulty hierarchy of the items could be verified as an indicator of the construct validity of the instrument.

#### Differential item functioning (DIF)

Finally, when comparing subjects by gender, four items (Item 1, Item 8, Item 9 and Item 15) showed differential item functioning. Items 1 (“My choice of clothing is affected”), 8 (“I feel embarrassed”), and 9 (“I feel frustrated”) with a DIF contrast of 0.70, 0.77, and 0.60, respectively, had a moderate to large impact (category C). Item 15 (“I avoid meeting new people”) with a DIF contrast value of − 0.48 was in category B, indicating a mild to moderate impact. It referred to avoiding new people due to the condition and was more symptomatic for men. Before testing for differential item functioning for age, we divided the sample into two subgroups. The discriminatory criterion was the medium age of 33 years in order to divide the sample in two groups, one representing younger patients and one composed of adults and older patients. Both groups accounted for approximately 50% of the original sample and were thus suitable for a valid comparison of the response behavior of these subgroups. When testing for differential item functioning for age three items were significant. Item 7 (“I feel nervous”) and Item 12 (“I worry about my future health”) were both in category B with a DIF contrast of − 0.54 and 0.53, respectively. Item 14 (“I worry about leaving sweat marks on things”) had a DIF contrast of − 1.12 (category C).

## Discussion

Applying CTT, we were able to reconfirm the two-factor structure of the HidroQoL. Moreover, structural validity was supported by further psychometric analyses using IRT and the Rasch model, which mainly reflected adequate fit. The dual structure of the HidroQoL allows on the one hand, the questionnaire to be interpreted as a measure of a unidimensional underlying construct, namely the quality of life of affected individuals. On the other hand, each domain can be further explored to investigate the differential impact of hyperhidrosis. DIF was found when controlling for gender and age for few items. However, this DIF could be explained in terms of content: for DIF by gender, the Items 1 (“My choice of clothing is affected”), 8 (“I feel embarrassed”), and 9 (“I feel frustrated”) showed a moderate to large impact. For all three items, there was a tendency for women to choose higher response categories, which can possibly be explained by the fact that women attach greater importance to external appearance than men, and consequently a more negative perception of it affects them more strongly [22]. Testing DIF by age, also resulted in three items having a significant impact (Item 7 (“I feel nervous”) and Item 12 (“I worry about my future health”), and Item 14 (“I worry about leaving sweat marks on things”)). For all three items, younger patients more likely selected the higher response options. This is especially not surprising for item 12, since the tendency to worry about one’s future health generally tends to decrease with age [23] and which might be indirectly related to item 7 and item 14, even though it is often underestimated how much importance young people attribute to their health [24]. According to Douglas and colleagues [25], there are two different types of DIF: adverse DIF occurs, when the probability of endorsing an item is different between groups because of artifacts in the measurement instrument, such as different understandings of the wording of items. This type of DIF represents a measurement error, since it is a bias in the measurement process. However, the second form of DIF does not represent a measurement error. Benign DIF occurs, when the varying probabilities of endorsing an item are governed by something other than the (dimension of the) construct measured by the instrument, such as belonging to a certain age group. Since this type of DIF reflects real differences in the underlying (dimension of the) construct and not different understandings of the wording for example, benign DIFs are not harmful to the measurement accuracy of the instrument. As described above, we could explain the DIFs between the different groups based on evidence and we were able to find real differences (e.g. greater importance to external appearance for women). Thus, we suppose that these reported DIFs can be categorized into the benign type and do not represent measurement errors. Thus, since all the relevant DIF could be explained, the DIF is unlikely to affect the reliability of the PROM.

The results of this study are in line with the findings presented by Kamudoni et al. [9], the initial validation of the HidroQoL, and those of Gabes et al. [10], who conducted further validation and clinical application of the HidroQoL. Gabes et al. [10] hereby used data from a randomized controlled phase III a trial. As the study progressed with the phase III b trial, this dataset was subsequently examined and analyzed in this work. A comparison of the results reported in the different studies on the measurement properties of the HidroQoL can be found in Table 5. It shows that the evidence for the good measurement properties was replicated at least once in different studies and that the findings of the three studies for each measurement property of the HidroQoL are complementary and mutually reinforcing.

**Table 5:** Overview of the measurement properties assessed for the HidroQoL

	Kamudoni, 2015: n = 260–595	Gabes, 2021: Phase III a, n = 171	Current study: Phase III b, n = 529
Structural validity	EFA and Rasch analysis only for scale development	X (2-factor CFA)	X (2-factor CFA and Rasch analysis)
Internal consistency (Cronbach’s alpha)	X	X	–
Reliability (ICCs)	X	X	–
Construct validity			–
Convergent/discriminant validity	X	X	–
Known-groups validity	X	X	–
Responsiveness	X	X	–

*HidroQoL* Hyperhidrosis quality of life index, *X* Assessed, *EFA* Exploratory factor analysis, *CFA* Confirmatory factor analysis, *ICCs* Intraclass correlation coefficients, *n* Sample size

### Strengths and limitations

With a sample size of *n* = 529 for the Rasch analysis, the requirements for a very good rating according to the COSMIN guidelines (sample size ≥ 200) were fulfilled [26]. Besides the large sample size, another strength of this study was the almost complete absence of missing data, reflecting a high motivation of the study participants to respond to the HidroQoL and indicating the ease of understanding and feasibility of the questionnaire. As limitations of this study one could mention the inclusion criteria, with patients reporting an HDSS of 3 or 4 only, indicating severe hyperhidrosis, as in the previous paper on the phase III-a part of the study [10]. Kamudoni and colleagues [8] did also include patients with an HDSS score ≥ 2, although eventually the majority of the sample were patients with an HDSS score of 3–4. Furthermore, in this study, DIF by country could not be assessed due to very large differences in the sample sizes of the various countries. Thus, in future studies, DIF by country or language should be investigated in order to broaden the validity evidence of the HidroQoL.

Additionally, significant DIF regarding age and gender was found. This can possibly affect the validity of the HidroQoL, since the response to the items showing DIF is governed by something other than the underlying construct health-related QoL. One common solution is to remove the items showing DIF from the questionnaire in order to preserve its validity. Nevertheless, the HidroQoL is a well-established and much used questionnaire in the clinical assessment of hyperhidrosis. Removal of items always needs to be balanced against maintaining a questionnaire in its original format enabling standardized assessment and comparability. For this reason, we refrained from removing these items right now. If future research also reports DIF in the same items, removal should be considered again since they can detrimentally affect the validity of the HidroQoL.

In this study, we could confirm the unidimensionality of the HidroQoL, as well as an underlying two-factor structure. This might be confusing, since both findings do not seem to align with each other. The HidroQoL as a whole scale is unidimensional (meaning that the HidroQoL has one underlying construct: health-related QoL) allowing the calculation of a sum score (confirmed by Rasch analysis and CFA). At the same time, it has two subscales (daily life activities and psychosocial domain) that are capturing different aspects of health-related QoL (confirmed by CFA). Both approaches aim for a different construct of hyperhidrosis impacts, and are not exclusive. They are based on different levels (two-factor solution: lower level, i.e. daily life activities or psychosocial impact versus unidimensionality: higher level, i.e. health-related QoL). The single factor solution (based on the Rasch analysis and CFA) seemed to be a more robust factor extraction approach than the two-factor solution (based solely on CFA), since in the first approach, the factor solution could be confirmed by CFA and PCA. Additionally, this study and the development study reported a correlation of the two subdomains of 0.651 and 0.645, respectively [8]. These correlations can be seen as an indicator that the unidimensionality might be more robust for this PROM than the two-factor solution. Nevertheless, Kamudoni also confirmed both solutions (one factor and two factors) with Rasch analysis and CFA, respectively [8]. Therefore, our results are in line with the previous research on this topic and both solutions could be confirmed twice. Nonetheless, the fit index we used to assess the CFA (SRMR) may not be the best one for this analysis. Unfortunately, the other fit indices did not pass the proposed thresholds. Thus, since only the SRMR reached the proposed threshold, the model has a reasonable fit to the data on only one aspect of the model’s fit. We suggest analyzing this two-factor solution in future studies and including different fit indices, such as CFI or TLI, in order to report more robust results regarding the two-factor structure. Additionally, in retrospect, other estimators than the ML might have been more suitable for ordinal data. Thus, we recommend for future studies calculating the CFA based on another estimator (i.e. weighted least square mean and variance adjusted (WLSMV) or diagonally weighted least squares (DWLS)), since this may lead to better fit indices and a better fit overall.

In summary, our study extends existing evidence on the measurement properties of the HidroQoL regarding structural validity, based on data from a large clinical trial in people with confirmed primary axillary hyperhidrosis. According to the COSMIN methodology, PROMs can be placed in the highest recommendation category A if evidence of sufficient content validity and at least low-quality evidence of sufficient internal consistency is provided. In addition, sufficient internal consistency requires at least low evidence of sufficient structural validity [26]. In this study, sufficient structural validity according to CTT and, for the first time, also according to IRT/Rasch could be confirmed. The criteria of content validity and internal consistency were demonstrated elsewhere [8, 10]. Thus, overall the HidroQoL questionnaire can be recommended for further use in clinical trials.

## Conclusion

Strong evidence supporting the conceptual structure and scoring approaches is fundamental to valid use of a PROM. The structural validity of the HidroQoL has been established in prior research. Using CTT and additional IRT/Rasch analyses, this study provided new evidence for the structural validity of the HidroQoL questionnaire using data from a phase III-b trial, thus helping to fill an important evidence gap for the HidroQoL. Overall, our findings support the dual structure of the HidroQoL allowing summation of scores to generate a single score, as well as calculation of separate domain scores for daily activities and psychosocial impacts. The findings are consistent with results of previous validation studies. Significant DIF was found which needs to be evaluated further in future studies.

## Data Availability

The data used and analyzed during the current study are available from the corresponding author on reasonable request.

## Appendix 1: COSMIN reporting guideline for studies on measurement properties of PROMs

**Table:**

General reporting recommendations relevant for all studies on measurement properties			
Item number	Item name	Item description	Location(s) reported in the manuscript
*Report section: title*			
T1	Patient reported outcome measure (PROM)	The name of the PROM instrument(s) (and version if relevant) being studied	Line 1 of the title
T2	Measurement property (MP)	What MPs are being studied or more generally, that MPs are being studied (if there are many properties being investigated, for example)	Line 1–2 of the title
T3	Study sample	General description of relevant study sample characteristics (e.g., condition of interest, language) and also any intervention or exposure (e.g., treatments) if applicable	Line 2 of the title
*Report section: abstract*			
A1	PROM	The name of the PROM instrument(s) (and version if relevant) being studied (i.e. the SF-36 or SF-12; language version) or if it concerns an item bank (e.g., PROMIS instruments). The type of instrument (e.g. a self reported questionnaire or interview)	Beginning of the section “Background” in abstract
A2	Measurement property	What MPs are being studied or more generally, that MPs are being studied (if there are many properties being investigated, for example)	Middle to end of the section “Background” in abstract
A3	Design	The type of study being used to test the properties (e.g., testretest design, longitudinal study, cohort, cross sectional, case series, randomized etc.). Other details of the study design if relevant (intervention/exposure, description of comparison instruments, outcomes other than PROMs)	Section “Methods” in abstract
A4	Sample	Inclusion/exclusion criteria. General description of relevant study sample characteristics (e.g., condition of interest, geographic location, language, other relevant demographic and baseline characteristics)	Beginning of the section “Results” in abstract
A5	Methods	A brief description of the methods for investigating each MP including statistical analyses	Section “Methods” in abstract
A6	Results	The main results for all MPs investigated reporting statistics for each result with measures of precision where appropriate	Section “Results” in abstract
A7	Discussion/conclusions	A brief description of the results in the context of existing evidence, main strengths and drawbacks and the need for future research on the PROM(s) investigated	Section “Conclusion” in abstract
*Report section: introduction*			
I1	Name and describe the PROM of interest	Specify the name, type, language, and version of the PROM being investigated and how it was developed. Describe the construct the PROM aims to measure and its subscales; describe the structure of the PROM (e.g., the number of factors, the number of items, scoring algorithm); describe relevant instructions (like time period), and number or type of response categories. State whether the PROM is based on a reflective or formative model. Note: This information may also appear in the methods section in greater detail	Middle to end of the section “Background”
I2	Target population	Describe the specific target population that the PROM was designed for. The authors need to provide the appropriate and necessary characteristics of this population	End of the section “Background”
I3	Citation for the original development of the PROM	The citation for the original development paper(s) should be provided and other highly relevant citations related to the quality of the specific PROM under investigation	Middle of the section “Background”
I4	State of knowledge & rationale	A description of the current scientific knowledge (what is known) regarding the MPs of? the PROM under investigation. The authors should provide a literature review or refer to a recent review of all existing evidence of the specific version (e.g., language, short 6 form) of the PROM and explain why the new study is necessary and important. The rational for the current proposed study should be given	Middle to end of the section “Background”
I5	Definitions	Specialized terms should be defined or explained	Not available
I6	Objectives and hypotheses	State the specific objective(s) of the research and hypotheses related to the specific PROM under investigation	End of the section “Background”
*Report section: general methods*			
GM1	Study design	State the key elements of the study design	Beginning of the section “Patients and Methods”
GM2	Participants	State how the participants were chosen; the inclusion and exclusion criteria. (e.g., if a PROM for a specific condition, then the eligibility and selection criteria should reflect this)	Beginning of the section “Patients and Methods”
GM3	PROM administration	An explicit description of how and when the PROM(s) were administered (e.g., in what setting) including data collection devices/system used (e.g. paper based, electronic administration/ePRO) should be provided	Beginning of the section “Patients and Methods”
GM4	Data collection procedures	Provide information about other data collection, exposure methods (e.g., allocation to interventions) and time points/follow-up points	Beginning of the section “Patients and Methods”
GM5	Power/sample size calculation	Provide a power calculation for all MP analyses. Alternatively, if a rule of thumb is used, state it and the source/citation	Not available
GM6	Statistical analyses	Statistical analyses and tests corresponding to all hypotheses or objectives for all MPs should be reported. Where appropriate, a cut-off for statistical significance should be reported (e.g., *p*-value less than 0.05). A description of all statistics to be used to estimate the magnitude and direction of effect should also be reported, together with measures of variability or precision. Report statistical package used	Section “Data analysis”
GM7	Missing data	State approaches or plan for dealing with missing data	Section “Distribution of responses” in “Patients and Methods”
GM8	Post hoc analysis	The report should specify analyses that used data after the data collection period concluded (i.e., if the analyses were post hoc; secondary data analyses) and describe the rationale for any post hoc analyses	Not available
*Report section: general results*			
GR1	Missing data	The amount and reasons for missing data should be explained for all analyses for all PROMs (or other outcome measurement instruments) and relevant groups	Section “Distribution of responses” in “Results”
GR2	Participant/patient characteristics	The study patients’ characteristics should be described, including baseline PROM scores	First abstract in the section “Results”
GR3	Sample size	If one study contained analyses using different sample sizes, the authors should report the sample size for each analysis	First line in the section “Results”
*Report section: discussion*			
D1	MP evidence	Per measurement property the authors should compare the result to the criteria for good measurement properties (e.g., COSMIN criteria) [27], and determine if the specific MP is sufficient or not. Note: This information may also appear in the results section in greater detail in a table for example	Last abstract before the section “Strengths and limitations”, Table 5
D2	Practical relevance	The authors need to discuss the practical relevance of the findings	Last abstract of the section “Strengths and limitations”
D3	Strengths and limitations	Strengths and limitations of the study should be discussed. For example, discuss if there were any significant potential biases in the study that could have impacted the results	Section “Strengths and limitations”
D4	Generalizability	Generalizability issues related to the PROM results should be discussed. For example, discuss if the results could be generalized to other populations given the sample studied	Last abstract before the section “Strengths and limitations”, Table 5
D5	Instrument changes	Discuss the need for modifications to the existing PROM or new 7 PROM development. If you conclude that one of the measurement properties is insufficient, you could suggest some modification, or if it is really poor, you could suggest stopping use of the PROM (in the specific population or in general)	Not available
D6	Future Research	Report specifically the type of research needed to answer new questions arising out of these findings for the particular MP and PROM investigated	Middle abstract of the section “Strengths and limitations”
*Report section: conclusions*			
C1	Conclusions	State the overall conclusions for each MP and of the use PROM investigated	Section “Conclusion”
*Report section: other information*			
O1	Conflict of interest	State any relevant conflict of interest related to the PROM under investigation (e.g., an author being the PROM developer, funding body etc.)	See Title Page

**Table:**

Specific reporting recommendations for studies on structural validity			
Item number	Item name	Item description	Location(s) reported in the manuscript
SV1	Factor analyses: Classical Test Theory (CTT) PROMs	Report details of the methods and results for any exploratory or confirmatory factor analyses. State the rational for any explorative factor analyses (e.g., no clear a priori hypotheses). For CFA, describe and justify the factor structure of tested models. Methods and results for checking of the assumptions should be described, the method of estimation, goodness-of-fit statistics and cut-off points for good model fit, including factor loadings of best-fitting model	Section “Using CTT: Confirmatory factor analysis” in “Patients and Methods” and in “Results”
SV2	Item Response Theory (IRT) analyses	Type of IRT/Rasch model should be reported. Also report the method of estimation, methods and results for checking of the assumptions (unidimensionality (see factor analysis), local dependency (e.g., residual correlations), monotonicity; (e.g. Mokken scaling), goodness-of-fit statistics, and cut-off points for goodness of item/model fit, and all item parameters	Section “Using IRT: Analysis of the response categories and performing Rasch analysis” in “Patients and Methods” and in “Results”

*COSMIN* COnsensus-based Standards for the selection of health measurement instruments, *PROM* Patient-reported outcome measure

## Abbreviations

CFA: Confirmatory factor analysis
CFI: Comparative fit index
CI: Confidence interval
COSMIN: Consensus-based Standards for the selection of health Measurement INstruments
CTT: Classical test theory
DIF: Differential item functioning
DLQI: Dermatology life quality index
EFA: Exploratory factor analysis
HDSS: Hyperhidrosis disease severity scale
HH: Hyperhidrosis
HidroQoL: Hyperhidrosis quality of life index
HQ: Hyperhidrosis questionnaire
ICCs: Item characteristics curves
IRT: Item response theory
MNSQ: Mean squares
PCA: Principal component analysis
PROMs: Patient-reported outcome measures
PSI: Person separation index
QoL: Quality of life
RMSEA: Root mean square error of approximation
SCI: Sweating cognitions inventory
SD: Standard deviation
SRMR: Standardized root mean square residual
TLI: Tucker–Lewis index
